# Analysis of Drugs Prescribed to Elderly Patients in a Tertiary Health Care Center in Raipur, Central India: An Observational Study

**DOI:** 10.7759/cureus.52770

**Published:** 2024-01-23

**Authors:** Yogendra Keche, Nitin R Gaikwad, Preetam N Wasnik, Keshao Nagpure, Md Sabah Siddiqui, Apoorva Joshi, Suryaprakash Dhaneria, Gevesh Dewangan, Jhasaketan Meher, Pranita Das

**Affiliations:** 1 Pharmacology, All India Institute of Medical Sciences, Raipur, Raipur, IND; 2 General Medicine, All India Institute of Medical Sciences, Raipur, Raipur, IND; 3 General Medicine, All India Institute of Medical Sciences, Nagpur, Nagpur, IND; 4 Pharmacology and Therapeutics, Ruxmaniben Deepchand Gardi Medical College, Ujjain, IND

**Keywords:** therapeutics of elderly, chronic disease prescription analysis, drugs and elderly, prescription analysis, geriatrics

## Abstract

Background

Most elderly patients suffer from multiple diseases and are on multiple drugs for treatment. Polypharmacy in the elderly, physiological changes with old age, changes in the pharmacokinetics and pharmacodynamic effects of many drugs, and newer drug prescription trends for diseases like diabetes and cardiovascular disease make drug prescribing in the elderly more difficult. There are many chances of drug-drug interactions with easily available over-the-counter (OTC) medications. To prevent the irrational use of drugs in the elderly, there is a need for prescription analysis studies. Prescription analysis studies will help in finding errors in prescriptions and also change trends in the use of medication among the elderly.

Methodology

This cross-sectional observation study was conducted on 234 elderly patients to investigate medicine use patterns among the geriatric patients attending the Medicine Outpatient Department in a tertiary care teaching hospital. Drug data were collected from the study participants after obtaining written informed consent and analysed, including demographic details, personal history, disease history, and details of the drug, including the generic name of the drug, dose and duration of therapy, and prescription pattern. The proportions of drugs prescribed for different diseases were analysed. Also, the drugs were analysed as per their pharmacological profiles.

Results and interpretation

A total of 1298 drug prescriptions were analysed in this study. Of the total participants, 60.26% were male, 35% were unemployed, 53% were retired, and 27% were taking OTC herbal medications. Most of the patients included in this study were suffering from diabetes, hypertension, and other comorbidities. Telmisartan and telmisartan in combination with other drugs were the most commonly encountered prescription drugs, i.e., 24% among the cardiovascular drugs. Aspirin and statins alone or in combination were the most commonly encountered prescriptions, i.e., 27.88% of the drugs used for prophylaxis of cardiovascular diseases.

Conclusion

This study showed a prescription pattern for the elderly and highlighted precautions to be taken with some of the prescribed drugs. As polypharmacy is observed with elderly prescriptions, possible drug interactions must be taken into account. Regular prescription analysis of drugs prescribed to the elderly will help in the appropriate and rational use of drugs.

## Introduction

India's elderly population is growing fast; as of now, 10.1% of the country's population is over 60 years of age. This percentage is expected to increase to 11.4% by 2026. The population aged 60 and over is expected to rise from 101.5 million in 2011 to 227.4 million in 2036 as a result of an increased lifespan [1]. Across all age groups, the prescription of medications is a crucial component of health care. Because of age-related physiological changes, altered pharmacokinetics, and altered pharmacodynamic effects of many drugs, prescribing for the elderly is always difficult. Furthermore, as older patients are excluded from clinical trials, information regarding the efficacy and safety of numerous medications frequently remains limited [2-4].

Numerous illnesses like diabetes mellitus, hypertension, Alzheimer's disease, Parkinson's disease, vascular dementia, stroke, visual impairment, cataracts and macular degeneration, dietary issues, and diminished financial resources result in decreased dosage compliance among the elderly [5]. There is a decline in renal function with ageing. The biomarkers of renal function are altered by malnutrition, a low-protein diet, and diminished muscle mass in elderly people. Therefore, normal-appearing urea and creatinine levels underestimate the severity of renal disease in the elderly [6-8]. Important alterations connected with the ageing process include, for example, forgetting to take one's medication due to cognitive and memory decline associated with a vascular or other disease. Due to lost income and higher costs, sickness causes economic strain. Until proven otherwise, all symptoms in an older patient are presumed to be adverse medication reactions. The elderly have a high potential for both beneficial and adverse drug effects [5]. Polypharmacy in the elderly leads to various adverse drug interactions, which necessitate the modification of drug therapy in them to avoid adverse drug interactions [9].

Correct use of medication is necessary to maximize therapeutic benefits while improving patients' quality of life. Drug prescriptions that are given haphazardly can lead to dangerous and ineffective treatment, medication non-adherence, greater rates of mortality and morbidity, disease progression, and higher expenditures on healthcare. Identifying the form, extent, and intensity of irrational drug use is a critical first step toward improvement in the rational use of medicines or limiting irrational medicine use [10,11].

According to a study conducted in Indonesia by Puspitasari et al., the most often prescribed antimicrobials in primary care settings were amoxicillin, chloramphenicol, ciprofloxacin, clindamycin, and oxytetracycline, while the most commonly prescribed other drugs were paracetamol, chlorpheniramine maleate, amlodipine, vitamin B complex, and glyceryl guaiacolat [12]. Abdulah et al. also noted similar results in another study conducted in Indonesia [13]. This study was carried out to gain more insight into the prescription pattern of drugs used for the elderly at a tertiary care teaching hospital in India.

## Materials and methods

This was a cross-sectional observation study for prescription analysis of drugs prescribed to elderly patients for chronic diseases. Patients aged above 65 years of age who attended the Medicine Outpatient Department (OPD) of All India Institute of Medical Sciences, Raipur, India, and who were on two or more drugs for chronic diseases were included in this study. Severely ill patients were excluded from this study. The study was approved by the Institute Ethics Committee, All India Institute of Medical Sciences, Raipur, India (approval number: 780/IEC-AIIMSRPR/2019, dated September 23, 2019). The study is registered with the Clinical Trial Registry of India (CTRI/2020/01/022852) [14].

Data collection

The data for this study were collected from 234 elderly patients. The drug data used in this analysis was already collected under the previously published study by the authors [15], which was unused earlier. Drug data were collected from the study participants after obtaining written informed consent from each participant. Collected data included demographic details, dietary history, personal history, history of constipation, peptic ulcer disease, and details of the drug, including the generic name of the drug, dose and duration of therapy, and prescription pattern. The number of drugs prescribed in every prescription was taken into account to calculate the incidence of polypharmacy.

Statistical analysis

An analysis of prescribed drug data and other factors in 234 elderly patients was carried out. Data were expressed as numbers, proportions, or the mean and standard deviation as applicable. Descriptive statistics were applied for analysis. All data was analyzed by use of Microsoft Excel (Microsoft Corporation, Redmond, Washington, United States).

## Results

The data of 234 elderly patients was analysed in this cross-sectional observational study, which included 1298 prescriptions. The average number of drugs prescribed per prescription was 5.92 ± 2.31 as a single drug and 2.15 ± 1.41 as fixed-dose combinations.

The mean age of the elderly patients in this study was 68.70 ± 5.76, and male patients were more (n=141, 60.26%). Forty patients (17.09%) were illiterate, 82 (35.04%) patients were unemployed, and 124 (53%) were retired from their jobs. A total of 171 (73%) elderly patients had an income of less than Rs. 5,000 per month. In the study population, 18 (7.96%) were alcoholics and 56 (23.93%) were smokers. A total of 81 (35%) had a history of constipation, 25 (11%) had a history of peptic ulcer disease, and 62 (27%) were taking over-the-counter (OTC) herbal medications (Table 1).

**Table 1:** Demographic data of study participants (N=234) OTC: over the counter; INR: Indian Rupee Data given as frequency (percentage) unless otherwise marked.

Demographic parameter	Value
Age (years), mean ± SD	68.70 ± 5.76
Sex, n (%)	
Male	141 (60.26)
Female	93 (39.74)
Education status, n (%)	
Illiterate	40 (17.09)
Primary School	55 (23.50)
Secondary School	70 (29.91)
Graduate	49 (20.94)
Postgraduate	20 (8.54)
Occupation status, n (%)	
Unemployed	82 (35.04)
Govt service	2 (0.85)
Private sector	20 (8.54)
Retired	124 (52.99)
Any other	6 (2.56)
Monthly income (INR), n (%)	
Less than 4999 per month	171 (73.08)
5000-9999	8 (3.42)
10000-49999	47 (20.09)
50000-100000	4 (1.71)
>100000 – 04	4 (1.71)
History of diet, n (%)	
Mixed diet	95 (40.60)
Vegetarian diet	139 (59.40)
Adequate protein in diet	204 (87.18)
Personal History, n (%)	
Alcohol	18 (7.69)
Smoking	56 (23.93)
History of constipation	81 (34.62)
History of acute peptic disease	25 (10.68)
Taking herbal OTC medications	62 (26.50)

Of the elderly in this study, 21 were suffering from diabetes mellitus (DM) (8.97%), 64 had DM with hypertension (27.35%), and 59 had DM with other comorbidities (25.21%). Thirty-one (13.25%) had only hypertension and 42 (17.95%) had hypertension with comorbidities (Table 2).

**Table 2:** Diagnoses of study participants (N=234)

S.No.	Diagnoses	Frequency (Percentage)
	Diabetes mellitus	21 (8.97)
1	Diabetes with hypertension	64 (27.35)
2	Diabetes mellitus with hypertension and other co-morbidities like osteoarthritis, chronic kidney disease, benign hypertrophy of prostate, coronary artery disease, myocardial infarction, hypothyroidism, diabetic kidney disease, total knee replacement, rheumatoid arthritis, Parkinson’s disease, asthma, intervertebral prolapse of disk, cerebrovascular accident, systemic lupus erythematous, psychosis with dementia, retinopathy, Nephropathy, heart failure, fatty liver, chronic obstructive pulmonary disease, cholelithiasis	59 (25.21)
3	Hypertension	31(13.25)
4	Hypertension with other co-morbidities like Parkinson’s disease, hypothyroidism, chronic obstructive pulmonary disease, herpetic neuralgia ,osteoarthritis, coronary artery disease, left ventricular hypertrophy, migraine, aortic regurgitation, asthma, hemiparesis, acute coronary syndrome, piles, benign hypertrophy of prostate, vertigo, peptic ulcer, cerebrovascular accident, seizure, dyslipidemia, depression, cholestasis, myocardial infarction, polyarthritis, stroke, myasthenia gravis, rheumatoid arthritis, generalized pruritis	42 (17.95)
5	Bronchial asthma	2 (0.85)
6	Sepsis, chronic kidney disease, acute kidney injury, severe anemia	1 (0.42)
7	Old cerebrovascular accident with hemiparesis and seizure	2 (0.85)
8	Rheumatoid arthritis with interstitial lung disease, chronic pain, cervical spondylosis, intervertebral prolapse of disk	4 (1.71)
9	Post-menopausal syndrome	1 (0.42)
10	Hepatitis C virus infection, chronic liver disease with portal hypertension, ascites	1 (0.42)
11	Chorea	1 (0.42)
12	Coronary artery disease, Parkinson	1 (0.42)
13	Chronic liver disease with portal hypertension, subclinical hypothyroidism	1 (0.42)
14	Vertigo	1 (0.42)
15	Chronic obstructive pulmonary disease with tuberculosis	1 (0.42)
16	Post coronary artery bypass graft	1 (0.42)

Telmisartan (n=58, 9.40%) and telmisartan combined with other drugs (n=82, 13.29%) were the most commonly prescribed drugs for cardiovascular disease. Other drugs predominantly prescribed were beta-blockers (n=42, 6.81%), calcium channel blockers (n=78, 12.64%), diuretics (n=31, 5.02%), and angiotensin-converting enzyme inhibitors (ACEIs) (n=14, 2.27%). Aspirin and statins alone or in combination (n=172, 27.88%) were most commonly prescribed for the prophylaxis of cardiovascular diseases (Table 3).

**Table 3:** Drug prescribing pattern for cardiovascular diseases, renal diseases, and diabetes

S.No.	Name of the drugs	Frequency (percentage)
Drugs for cardiovascular and renal diseases (N=617)		
1.	Telmisartan	58 (9.40)
2.	Telmisartan and amlodipine or cinlidipine or metoprolol or nebivolol or chlorthalidone or hydrochlorothiazide	79 (12.80)
3.	Telmisartan and amlodipine and hydrochlorthiazide	2 (0.32)
4.	Telmisartan and cilnidipine and chlorothalidone	1 (0.16)
5.	Labetolol or bisprolol or carvedilol or nebivolol	13 (2.11)
6.	Metoprolol	29 (4.70)
7.	Ramipril	13 (2.11)
8.	Ramipril and hydrochlorothiazide or Cilnidipine and metoprolol	2 (0.32)
9.	Cilnidipine	22 (3.57)
10.	Aspirin	16 (2.59)
11.	Aspirin and atorvastatin or rosuvastatin	57 (9.24)
12.	Aspirin and clopidogrel	2 (0.32)
13.	Aspirin and rosuvastatin and clopidogrel	2 (0.32)
14.	Aspirin and atorvastatin and clopidogrel	4 (0.65)
15.	Dabigatran	1 (0.16)
16.	Atorvastatin	79 (12.89)
17.	Rosuvastatin	12 (1.94)
18.	Erythropoietin	2 (0.32)
19.	K bind sachet	2 (0.32)
20.	Spironolactone and torasemide or furosemide	9 (1.45)
21.	Nitroglycerine or Isosorbide mononitrate or Isosorbide dinitrate	8 (1.30)
22.	Furosemide or torasemide or chlorthalidone	22 (3.56)
23.	Nicorandil	11 (1.78)
24.	Amlodipine or Nifedipine	50 (8.10)
25.	Amlodipine and atenolol or hydrochlorothiazide	6 (0.97)
26.	Ranolazine	3 (0.49)
Drugs for Diabetes (N=262)		
27.	Insulin	24 (9.16)
28.	Metformin	49 (18.70)
29.	Glimepiride	29 (11.07)
30.	Glimepiride and metformin	53 (20.23)
31.	Metformin and voglibose or sitagliptin or teneligliptin or vildagliptin or gliclazide	27 (10.31)
32.	Metformin and glimepiride and pioglitazone	5 (1.91)
33.	Metformin and glimepiride and voglibose	4 (1.53)
34.	Teneligliptin	28 (10.69)
35.	Linagliptin or vildagliptin	3 (1.15)
36.	Empagliflozin or repaglinide	2 (0.76)
37.	Gliclazide	2 (0.76)
38.	Acarbose or voglibose	31 (11.83)
39.	Pioglitazone	5 (1.91)

Metformin (n=49, 18.70%) and glimepiride (n=29, 11.07%) were commonly used drugs for diabetes, either alone or in combination (n=89, 33.97). Teneligliptin (n=28, 10.69%) and alpha-glucosidase inhibitors like acarbose and voglibose (n=31, 11.83%) were also commonly used for the treatment of diabetes in the elderly (Table 3).

Clonazepam (n=14, 16.47%) was the most commonly prescribed drug for CNS diseases. Gabapentin and pregabalin alone or in combinations (n=28, 32.94%) were used for the treatment of neuropathies (Table 4).

**Table 4:** Drug prescribing pattern for central nervous system diseases, inflammation, and autocoids

S.No.	Name of the drugs	Frequency (percentage)
Sedatives, hypnotics, psychopharmacological agents (N=85)		
1.	Alprazolam or lorazepam	2 (2.35)
2.	Clonazepam	14 (16.47)
3.	Zolpidem	2 (2.35)
4.	Carbamazepine or oxcarbazepine	3 (3.53)
5.	Topiramate	1 (1.18)
6.	Valproate	1 (1.18)
7.	Gabapentin and nortriptyline	9 (10.59)
8.	Pregabalin	2 (2.35)
9.	Pregabalin and methylcobalamin	15 (17.65)
10.	Pregabalin and nortriptyline	2 (2.35)
11.	Donepazil and memantine	1 (1.18)
12.	Donepezil	3 (3.53)
13.	Levodopa and carbidopa	8 (9.41)
14.	Olanzapine or quetiapine	3 (3.53)
15.	Levosulpiride	1 (1.18)
16.	Amitriptyline	5 (5.88)
17.	Escitalopram or levetiracetam or sertraline	6 (7.06)
18.	Escitalopram and clonazepam	1 (1.18)
19.	Fluoxetine or paroxetine	2 (2.35)
20.	Taurine and acetylcysteine	1 (1.18)
21.	Tetrabenazine	1 (1.18)
22.	Trihexyphenidyl	1 (1.18)
23.	Pyridostigmine	1 (1.18)
Analgesics and anti-inflammatory drugs (N=45)		
24.	Aceclofenac and paracetamol	2 (4.44)
25.	Chlorzoxazone and ibuprofen and paracetamol	1 (2.22)
26.	Trypsin and chymotrypsin	1 (2.22)
27.	Etoricoxib	1 (2.22)
28.	Ibuprofen and paracetamol	1 (2.22)
29.	Indomethacin	3 (6.67)
30.	Mefenamic acid and dicyclomine	1 (2.22)
31.	Tramadol	6 (13.33)
32.	Tramadol and paracetamol	1 (2.22)
33.	Paracetamol	28 (62.22)
Antihistaminics and autocoids (N=8)		
33.	Cetirizine	2 (25)
34.	Cyproheptadine	2 (25)
35.	Betahistine hydrochloride	1 (12.50)
36.	Propranolol and flunarizine	1 (12.50)
37.	Cinnarizine or cinnarizine and dimenhydrinate	2 (25)
Corticosteroids (N=3)		
38.	Decadurabolin	1 (33.33)
39.	Prednisolone	2 (66.67)

Pantoprazole alone or in combination with domperidone (n=82, 64.57%) was most commonly prescribed for acid-peptic disease. Thyroxin (n=26, 100%) was most commonly prescribed for hypothyroidism, and vitamin preparations (n=118, 62.43%) were also most commonly prescribed to elderly patients (Table 5).

**Table 5:** Drug prescribing pattern for other drugs/disorders

S.No.	Name of the drugs	Frequency (percentage)
Gastro-intestinal disorders (N=127)		
1.	Pantoprazole	42 (33.07)
2.	Pantoprazole and domperidone	38 (29.92)
3.	Pantoprazole and levosulpiride or Esomeprazole and domperidone	2 (1.57)
4.	Omeprazole	8 (6.30)
5.	Omeprazole and tinidazole and amoxicillin	1 (0.79)
6.	Ranitidine	2 (1.57)
7.	Antacid syrups	4 (3.15)
8.	Isabghol husk or bisacodyl	3 (2.36)
9.	Milk of magnesia and liquid paraffin syrup	12 (9.45)
10.	Lactulose syrup	11 (8.66)
11.	Ondansetron	3 (2.36)
12.	Ursodeoxycholic acid	1 (0.79)
Endocrine diseases (N=26)		
13.	Thyroxin	26 (100)
Arthritis (N=15)		
14.	Hydroxychloroquine	9 (60)
15.	Ibandronic acid	2 (13.33)
16.	Methotrexate	4 (16.67)
Respiratory diseases (N=11)		
17.	Omalizumab	1 (9.09)
18.	Doxofylline	4 (36.36)
19.	Etophylline and theophylline	1 (9.09)
20.	Montelukast	2 (18.18)
21.	Montelukast and levocetirizine	3 (27.27)
Hypertrophy of prostate (N=22)		
22.	Dutasteride	1 (4.55)
23.	Tamsulosin	7 (31.81)
24.	Tamsulosin and dutasteride	8 (36.36)
25.	Silodosin	1 (4.55)
26.	Solifenacin	5 (22.73)
Miscellaneous drugs (N=189)		
27.	Disodium hydrogen citrate syrup	3 (1.59)
28.	Cough syrup	3 (1.59)
29.	Glycerol syrup	1 (0.53)
30.	Sulfasalazine	6 (3.17)
31.	Febuxostat	3 (1.59)
32.	Flavoxate hydrochloride	1 (0.53)
33.	Iron and folic acid	7 (3.70)
34.	Herbal drugs	2 (1.06)
35.	Topical formulations	38 (20.11)
36.	Vitamins	118 (62.42)
37.	Antimicrobials	7 (3.70)

## Discussion

In this cross-sectional observational study, the average number of drugs prescribed per prescription was 5.92 ± 2.31 for single drugs and 2.15 ± 1.41 for fixed-dose combinations. The mean age of the elderly patients in this study was 68.70 ± 5.76, and 141 (60.26%) patients were male. In a study carried out at Pudducherry, India, the average number of drugs prescribed per prescription was 9.4 ± 0.31, which was higher than our study [16], but in two other studies, the average number of drugs per prescription was comparable with our study [17,18]. The male preponderance that was seen in our study was similar to other studies [16-18]. The average number of drugs per prescription is an important index of the scope for review and intervention in prescribing practices.

In the current study, around 82 (35%) patients were unemployed, 124 (53%) were retired, and 171 (73%) had an income of less than Rs. 5,000 per month. The majority of patients in a study by Lourdu et al. were found to be retired from their jobs, making them dependent on their families for financial support [16].

A total of 81 (35%) of the elderly had a history of constipation in the present study. The history of constipation is important, as the majority of patients in this study were suffering from hypertension or other cardiovascular diseases. Calcium channel blockers (CCBs) like amlodipine and nifedipine are routinely prescribed to these patients, and it was observed in a study conducted by Saha et al. that there is an increased risk of developing constipation with the use of these drugs [19]. Hence, precautions need to be taken while prescribing CCBs to the elderly, especially those who have a history of constipation. Twenty-five (11%) had a history of peptic ulcer disease in the current study, but the prescription encounters were more for pantoprazole alone or in combination. As per STOPP (Screening Tool of Older Persons' Prescriptions)/START (Screening Tool to Alert to Right Treatment) criteria, if proton pump inhibitors (PPI) are prescribed for uncomplicated peptic ulcer disease at full therapeutic dosage for more than eight weeks, there is usually a need for dose reduction, earlier discontinuation, or H2 antagonist maintenance therapy [20]. PPI is to be prescribed carefully if therapy goes beyond eight weeks.

About one-fourth of elderly patients in the current study were taking OTC herbal medications. Scherf-Clavel, in a review of OTC medications, identified the groups that were frequently involved in drug-drug interactions (DDIs) as NSAIDs, food supplements, antacids, PPIs, H2 antihistamines, laxatives, antidiarrheal drugs, and herbal drugs [21]. To avoid adverse drug interactions and appropriate prescriptions of drugs in elderly patients, STOPP/START criteria and Beers criteria can be used [15].

In the current study, the elderly were suffering from diabetes mellitus, hypertension, and other comorbidities. There are regional differences in the prevalence of diseases among the elderly. For example, in Jammu and Kashmir, there is a higher prevalence of cardiovascular, respiratory, endocrine, and gastrointestinal diseases [16]. In contrast, a study conducted in south India found that respiratory conditions like bronchial asthma, chronic bronchitis, and COPD were more common, and drugs used for the treatment of these diseases were more commonly prescribed [17]. In a study in Karnataka, the commonly involved target organ system that necessitated the need for admission was the respiratory system followed by neurological and cardiovascular systems [18].

Telmisartan by itself or in combination with other drugs, beta-blockers, calcium channel blockers, diuretics, and ACE inhibitors were more commonly prescribed for the treatment and prophylaxis of cardiovascular diseases in our study. Aspirin and statins alone or in combinations were most commonly prescribed for prophylaxis of cardiovascular diseases. Metformin, glimepiride alone or in combinations, teneligliptin, and alpha-glucosidase inhibitors like acarbose and voglibose were also commonly used for the treatment of diabetes in the current study. Pantoprazole alone or in combination with domperidone for acid peptic disease and thyroxin for hypothyroidism and vitamin preparations were also most commonly prescribed to the elderly patients in the current study. Prescription patterns are different in studies carried out in different parts of India. Drugs that were prescribed to the elderly in other studies included Deriphyllin most commonly, paracetamol, ranitidine, amlodipine, vitamin B12, furosemide, metformin, dexamethasone, doxycycline, ceftriaxone, alprazolam, salbutamol, ferrous sulphate, mannitol, and spironolactone [16,18].

Benzodiazepines like clonazepam were more commonly prescribed to the elderly than z-hypnotics in the current study. In a study carried out in Taiwan that analysed the data on the 10-year use of z hypnotics, it was found that there was an increase in the use of z hypnotics in Taiwan [22]. Age has been identified in multiple studies as a major risk factor for falls and fractures in the elderly population, particularly those associated with z-hypnotics [23,24]. There is insufficient evidence about the safety and efficiency of z-hypnotics in the elderly population. Exposure to zolpidem has been linked to an elevated risk of fracture in the senior population, with individuals aged over 85 years having the highest risk.

Additionally, benzodiazepines increase the risk of drug misuse, dependency, tolerance, falls, and fractures [25,26]. To avoid adverse events related to hypnotics, appropriate use of these drugs needs to be carried out. Gabapentin and pregabalin alone or in combination were used for the treatment of neuropathies in the current study.

Drug utilization and prescription analysis studies are helpful in finding the prescription pattern and appropriate and rational use of drugs in the elderly. Apart from drug utilization studies and drug appropriateness studies, there is a need for studies on the quality of life of elderly patients, which is affected greatly due to various factors like comorbidities, mental status, and physical activity [27].

Strengths and limitations

This study provides insight into the current trends of drug prescription among the elderly in the Indian population for chronic diseases. Limitations include the fact that this is a single-centre study, and the findings of this study cannot be generalised to other parts of the country. As this study involves only the analysis of drugs prescribed to the elderly in terms of names of the drugs prescribed, proportions of drugs, and diagnoses of the disease, the inappropriateness of the prescription cannot be commented on.

## Conclusions

Polypharmacy was observed in this study and shows that there is a need for watchfulness for DDIs. As cardiovascular diseases and diabetes, with or without comorbidities, were more commonly observed, drugs used for treatment and prophylaxis of these ailments were seen to be commonly prescribed in this study. As benzodiazepines are prescribed to elderly people, care for falls and fractures needs to be taken, and instructions must be given to the patient to avoid falls. PPIs should not be prescribed rampantly to the elderly, and similarly, vitamin preparations should be prescribed judiciously. Regular prescription analysis of drugs prescribed to the elderly will help in the appropriate and rational use of drugs and will also help observe changes in prescription trends in the elderly.
